# Supplementary material for: The CaMKII D135N mutation blocks kinase activity and reduces GluN2B binding
Source: Mol Pharmacol. 2026 May 7;108(5-6):100127. doi: 10.1016/j.molpha.2026.100127 (PMC13727650; doi:10.1016/j.molpha.2026.100127)
Supplement: Supplementary file 1 [file mmc1.pdf]

Supplementary Figures for:

Matthew E. Larsen, C. Madison Barker, Raul Satoshi Vargas, Steven J. Coultrap, and K. Ulrich Bayer, "**The CaMKII D135N mutation blocks kinase activity and reduces GluN2B binding**", Molecular Pharmacology

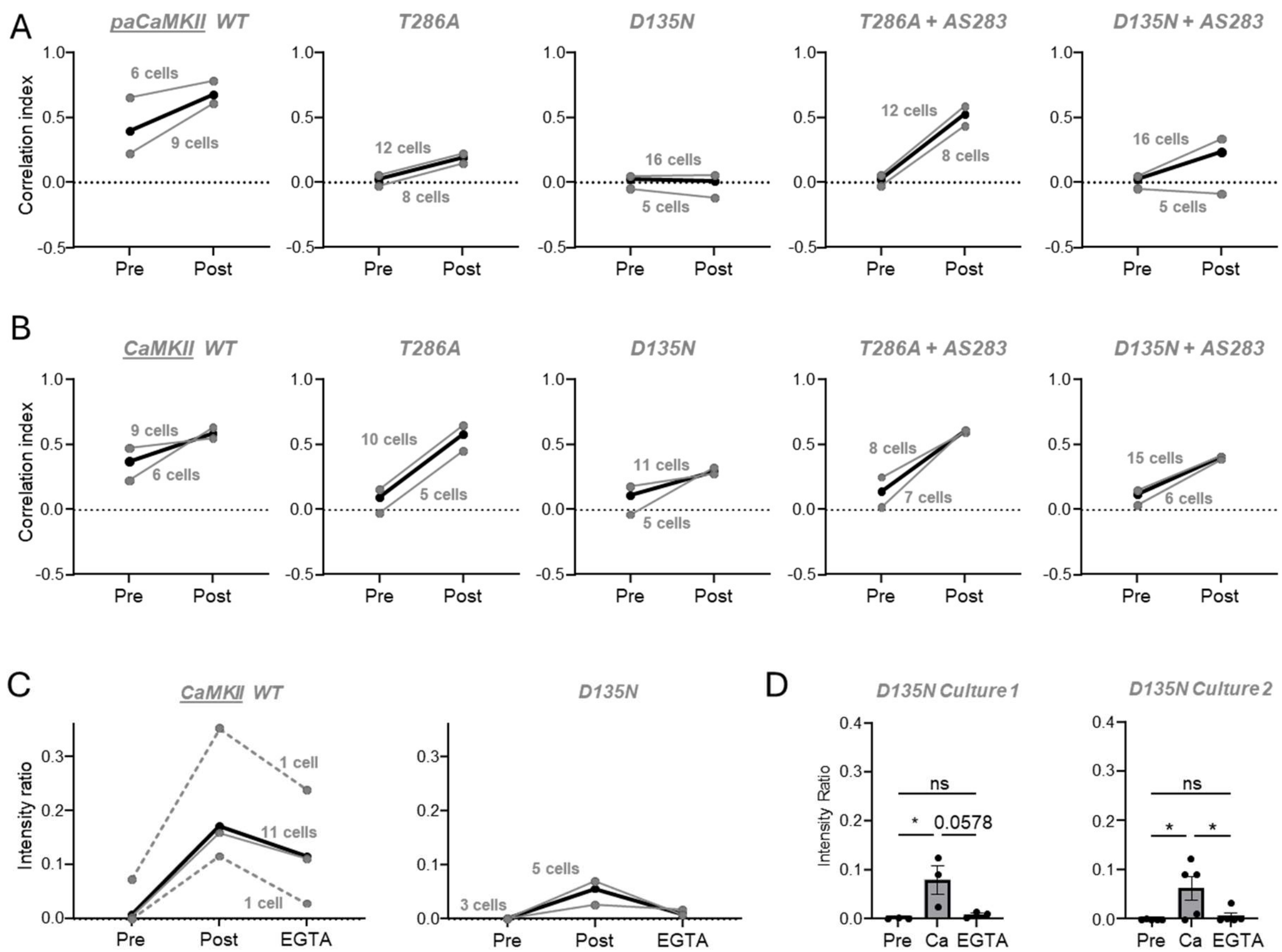

Supplemental Fig. S1. Contribution of the independent cultures to the overall observed CaMKII movement in HEK cells. The overall CaMKII under the different conditions is indicated by the thick black line that connects the overall means of co-localization with GluN2B before and after stimulation (black circles). The corresponding means from the individual independent cultures are indicated by gray circles connected by thinner gray lines and the number of cells (n) analyzed per individual culture is indicated.

(**A**) Contributions of two independent cultures to the overall light-induced co-localization of the photoactivatable paCaMKII to GluN2B (related to Figure 3).

(**B**) Contributions of two independent cultures to the overall co-localization of the CaMKII to GluN2B induced by Ca^{2+} signals triggered by stimulation with ionomycin (related to Figure 4).

(**C**) Contribution of two to three independent cultures to the overall Ca^{2+}-induced co-condensation of CaMKII with the non-targeted GluN2B C-tail and test for persistence after chelation of Ca^{2+} with EGTA (related to Figure 5). While the overall results for CaMKII WT in this study are dominated by one single culture, the same results have been obtained for this control condition in two other recent studies from our lab (Rumian et al., 2023 and Brown et al., 2025).

(**D**) Each of the two independent cultures for co-condensation of the D135N mutant with GluN2B shows a similar statistical outcome as the overall results in Figure 5D. Error bars indicate SEM. The values are indicated with numerical value or with ns ($p > 0.1$) or * ($p < 0.05$).

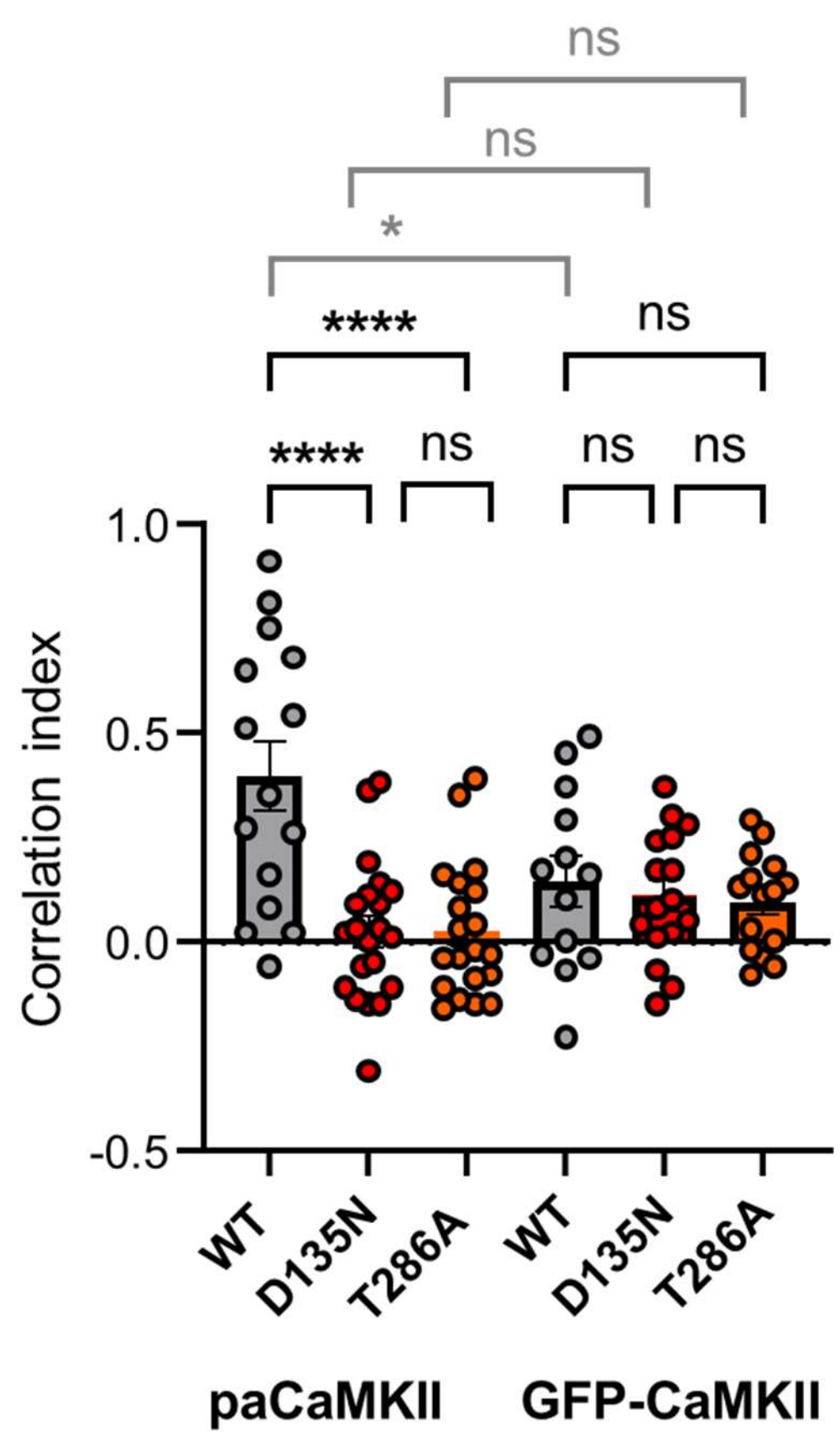

Supplemental Fig. S2. Basal GluN2B colocalization of the different CaMKII constructs prior to stimulation. The basal colocalization of the photoactivatable paCaMKII appears to be increased compared to all other constructs (n=15,21,20,13,16,15 cells, one-way ANOVA with Tukey's multiple comparisons test), but this apparent increase is due to photoactivation during the acquisition of the z-stack for the "basal" condition (as evident by the increased colocalization in the last image of the z-stack compared to the first image of the stack; see Fig. 3A,B). *p<0.5, ****p<0.0001. Error bars indicate SEM.
